# Ultrastructural analysis of Lyophilized Human Spermatozoa

**DOI:** 10.5935/1518-0557.20210028

**Published:** 2021

**Authors:** Renata de Lima Bossi, Marcelo Cabral, Monica Oliveira, Sávia Lopes, Rodrigo Hurtado, Marcos Sampaio, Selmo Geber

**Affiliations:** 1 Department of Obstetrics and Gynaecology of the Medical School, Universidade Federal de Minas Gerais, Belo Horizonte, MG, Brazil; 2 ORIGEN, Center for Reproductive Medicine, Belo Horizonte, MG, Brazil; 3 Faculty of Pharmacy, Universidade Federal de Minas Gerais, Belo Horizonte, MG, Brazil

**Keywords:** lyophilization, human semen, cryopreservation, transmission electron microscopy

## Abstract

**Objective:**

Lyophilization is potentially more practical and cost-effective alternative for sperm preservation. However, there are no studies that evaluate the ultrastructure of human spermatozoa after lyophilization. Therefore, the aim of our study was to evaluate the ultrasctructure of lyophilized spermatozoa using Transmission Electron Microscopy.

**Methods:**

From a total of 21 donated seminal samples, 30 aliquots were originated and divided into two aliquots so that one could have been submitted to cryopreservation/thaw and the other for lyophilization/rehydration. The liquefied aliquots were homogenized at room temperature. Samples assigned for cryopreservation were placed in straws and samples assigned for lyophilization were placed in the appropriate vials. Cryopreservation samples were placed at -30oC for 30 minutes subsequently for 30 minutes at vapour phase and then plunged into liquid nitrogen. Lately, were warmed in water bath at 37oC for 10 minutes followed by 10 minutes centrifugation. The pellet was resuspended and analysed in a Makler chamber. The semen vials assigned for lyophilization were loaded into a pre-fixed freeze-drying chamber. Following lyophilization, vials were removed from the freeze-drying chamber and kept at 4oC until rehydration. TEM was performed after rehydration and thawing. Sperm samples were fixed, rinsed in buffer, post fixed and dehydration was carried out in escalating concentrations of alcohol solution, acetone and then, embedding in Epon resin. Ultrathin sections were stained and examined in a Transmission Electron Microscope.

**Results:**

Analysis of sperm after freezing/thawing using Transmission Electron Microscopy showed lesions to the midpiece, with some mitochondria degeneration and random rupture of plasma membrane. In the head, we identified intact plasma membrane, nucleus and acrosome, as in the flagellum all main structures remained intact including the plasma membrane, the longitudinal columns of dense fibers and the semicircular fibers. Analysis by Transmission Electron Microscopy showed that spermatozoa heads had ruptured plasma membranes, absence of acrosomes, nuclei with heterogeneous and decompressed chromatin. Mitochondria were deteriorated in the midpiece. Longitudinal columns of dense fibers were absent in the flagellum. Axonemes, in cross-sections, were disrupted with disorganized structures.

**Conclusions:**

To our knowledge, our study demonstrated, for the first time, the structure of the human spermatozoa after lyophilization using Transmission Electron Microscopy. The use of a fixed lyophilization protocol with media containing cryoprotectants might explain the damage to the structures. More studies are necessary to improve the results of sperm lyophilization. In the future, the use of lyophilization of spermatozoa might reduce the costs of fertility preservation, since there will be no need for storage space and transportation is simpler.

## INTRODUCTION

Cryopreservation of human spermatozoa is a widely used approach for male fertility preservation in cancer patients, as well as in other non-malignant conditions. It is also frequently applied for patients who will undertake fertility treatments using donor semen, patients who need to store spermatozoa for non-medical reasons and for azoospermic patients. Patients who have undergone testicular or epididymal sperm extraction and wish to avoid repeated surgical procedures may also benefit from this technique (Anger *et al*., 2003).

There are a few different liquid nitrogen-based methods for sperm cryopreservation such as slow freezing (Royere *et al*., 1996; Isachenko *et al*., 2012; Nallella *et al*., 2004), vitrification (Isachenko *et al.,* 2005; Kuwayama *et al*., 2005; Vajta & Nagy, 2006; Aizpurua *et al*., 2017; Shah *et al*., 2019; O’Neill *et al*., 2019), cryopreservation in empty zona pellucida (Cohen *et al*., 1997; AbdelHafez *et al*., 2009) and Cell Sleepers (Endo *et al*., 2012; Coetzee *et al*., 2016; Herbemont *et al*., 2019) and Sperm VD (Berkovitz *et al*., 2018; Li *et al*., 2019). Mild harmful effects on sperm structure and function may occur, regardless of the utilized cryopreservation technique. Thermal shock may induce intra and extracellular ice crystals formation, besides osmotic shock caused by excessive dehydration, which may also promote sperm death. Furthermore, the use of liquid nitrogen imposes some operative drawbacks such as the considerable storage space needed, transportation difficulties, risk of cross contamination of the samples, and maintenance costs (Clarke, 1999; Bielanski *et al*., 2003; Morris, 2005; Gianaroli *et al*., 2012). Lyophilization of spermatozoa might be a helpful alternative to liquid nitrogen-based methods.

Lyophilization or freeze-drying is a sublimation process in which water goes from the solid directly to the gas phase, under high vacuum and low (below freezing point) temperature (Gianaroli *et al*., 2012). Since there is nearly no need for storage space and transportation is simpler and the cost is considerably reduced. Also, as lyophilization leads to virus inactivation, the risk of viral contamination is more likely eliminated (Unger *et al*., 2009). The feasibility of this method has been reported in animal studies, with live birth reports of viable offspring after intracytoplasmic sperm injection (ICSI) in mice (Wakayama & Yanagimachi, 1998; Kusakabe *et al*., 2001; Ward *et al*., 2003; Kawase & Suzuki, 2011; Kusakabe, 2019; Wakayama *et al*., 2017), rabbits (Liu *et al*., 2004; Mercati *et al*., 2020), rats (Hirabayashi *et al*., 2005; Hochi *et al*., 2008; Muneto *&* Horiuchi, 2011), horse (Choi *et al*., 2011; Restrepo *et al*., 2019), other domestic animals and endangered species (Kaneko *et al*., 2014; Olaciregui *et al.,* 2015; Anzalone *et al*., 2018; Lv *et al*., 2019).

In humans, only scarce data have been published so far. Kusakabe *et al*. (2008) described chromosome integrity on human sperm after lyophilization and injection into enucleated mouse oocytes. Gianaroli *et al*. (2012) unveiled that lyophilization of human sperm damages cell membranes, but it doesn’t affect DNA’s integrity and Zhu *et al*. (2016) observed head and tail membrane damage using scanning electron microscopy (SEM)

To our knowledge there are no published studies that evaluate the ultrastructure of human spermatozoa after lyophilization and Transmission Electron Microscopy (TEM) is a valid tool for such evaluation. Therefore, the aim of our study was to evaluate the ultrasctructure of lyophilized spermatozoa using TEM.

### Material and methods

A total of 21 healthy men who had been subjected to assisted reproductive technology (ART) treatment from August 2014 to August 2015 donated their remaining semen sample for this study. All donors read and signed an informed consent form. The study was approved by the Research Ethics Committee of the Universidade Federal de Minas Gerais (COEP/UFMG - No 743.984). Semen samples were classified as normozoospermic according to World Health Organization (WHO) criteria.

### Semen preparation

After ejaculation, semen samples were allowed to liquefy at room temperature for 30 minutes and were classified for count, motility and morphology, according to the World Health Organization (WHO, 2010) criteria. After selection for ICSI, the remaining volume was divided into two aliquots: one for cryopreservation/lyophilization and the other for cryopreservation, as a control.

The liquefied aliquots were homogenized with Freeze Medium (Irvine Scientific, USA) or Sperm Freeze Solution (Vitrolife, Sweden), both at room temperature in 1:1 proportion. Samples assigned for cryopreservation were placed in 0,5 mL CryoBiosystem straws (IMV Technologies, France), and samples assigned for lyophilization were placed in the appropriate vials for freeze-drying.

### Cryopreservation and Thawing

Cryopreservation samples were placed at -30ºC for 30 minutes subsequently for 30 minutes at vapour phase and then plunged into liquid nitrogen. Cryopreserved sperm samples (control) were warmed in water bath at 37ºC for 10 minutes followed by 10 minutes centrifugation at 200 G with culture medium mHTF (Irvine Scientific, USA). The pellet was resuspended in 0,3 mL of mHTF and then analysed in a Makler chamber.

### Lyophilization and Rehydration

The semen vials assigned for lyophilization were loaded into a freeze-drying chamber (ModulyoD-115, Thermo Fisher Scientific) for 24 hours where temperature was maintained at -50ºC and pressure at 50-100 µbar. Following lyophilization, vials were removed from the freeze-drying chamber and kept at 4ºC until rehydration with 0.5 mL of water for embryo transfer (Sigma, USA) and analysed by optical microscopy.

### Ultrastructural analysis

Transmission Electron Microscopy was performed after completion of rehydration and thawing of lyophilized and frozen samples for ultrastructural analysis, at the Centro de Microscopia Eletrônica da Universidade Federal de Minas Gerais. Sperm samples were fixed for 24 hours at a 4ºC temperature in a solution containing 2.5% glutaraldehyde, 2% paraformaldehyde, and 0.1 M of phosphate buffer. After fixation, the specimens were rinsed in buffer, and post fixed in 2% osmium tetroxide (OsO_4_) for 2 hours at room temperature. Dehydration was carried out in escalating concentrations of alcohol solution (35%-100%), followed by acetone and then, embedding in Epon resin. Ultrathin sections (50-60 nm) were stained with lead citrate (Reynolds Solution) and examined in a Tecnai G2-12 (SpiritBiotwin FEI - 120 kV) Transmission Electron Microscope, operating at 80 kV (Oregon, USA).

## RESULTS

From a total of 21 donated seminal samples, 30 aliquots were originated and divided into two aliquots so that one could have been submitted to cryopreservation/thaw and the other for lyophilization/rehydration. The patients’ age ranged from 32 to 56 years (38.7±5.2 years) and the sexual abstinence ranged from 3 to 10 days (3.7±2.1 days). Seminal volume ranged from 1.5 to 4.6 mL (2.6±1.5 mL), pH ranged from 7.5 to 8.5 (8.02±0.06). Initial sperm concentration ranged from 20×10^6^ to the maximum of 176×10^6^ (75.74×10^6^±27.2). Progressive motility ranged from 28% to 89% (59%±18.52); non-progressive motility ranged from 0 to 20% (26.05%±6.22). Normal morphology ranged from 4% to 18% (7.95%± 2.1%). Normal chromatin ranged from 70% to 98% (90.3%±5.3%).

**Figure 1:**
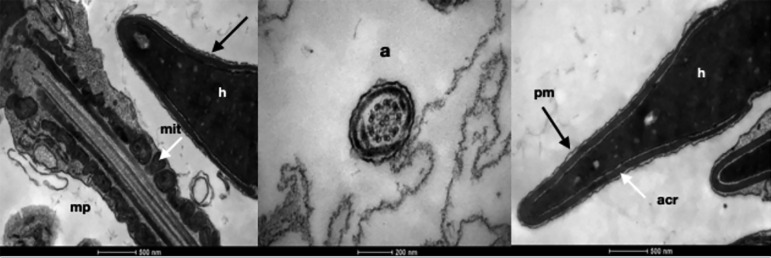
Transmission Electron Microscopy of Fresh Sperm: structure and organelles intact. a: axoneme, acr: acrossome, h: head, mit: mitochondria, mp: midpiece, pm: plasma membrane.

Fresh spermatozoa analysis using Transmission Electron Microscopy showed intact heads and plasma membranes, compact and homogeneous chromatin in the nuclei, physiological vacuoles, acrosomes with visible internal and external membranes and normal subacrosomic spaces. In the midpiece, mitochondria and external dense fibers were observed. It was also possible to identify the semicircular fibers and two longitudinal columns of dense fibers, alongside with the plasma membrane, constituting the flagellum. Transverse sections of the axonemes from the middle piece, showed the plasma membrane, mitochondria sheath involving the outer dense fibers, nine pairs of peripheral microtubules plus two pairs of central microtubules. The transverse sections of axonemes from the flagellum region showed plasma membrane involving the sheath of dense fibers, nine pairs of peripheral microtubules plus two pairs of central microtubules (Figure 1).

**Figure 2:**
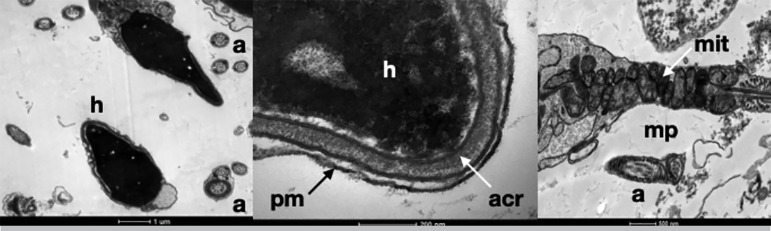
Transmission Electron Microscopy of Cryopreserved Sperm: structure intact. a: axoneme, acr: acrossome, h: head, mit: mitochondria, mp: midpiece, pm: plasma membrane.

Analysis of sperm after freezing/thawing showed concentration ranging from 5.6×10^6^ to 140×10^6^ (44.75×10^6^±35.08), progressive motility ranging from 1 to 58% (23.6%±14.45) and non-progressive motility ranged from 2 to 20% (10,65%±4.90). Transmission Electron Microscopy showed lesions to the midpiece, with some mitochondria degeneration and random rupture of plasma membrane. In the head, we identified intact plasma membrane, nucleus and acrosome, as in the flagellum all main structures remained intact including the plasma membrane, the longitudinal columns of dense fibers and the semicircular fibers (Figure 2).

**Figure 3:**
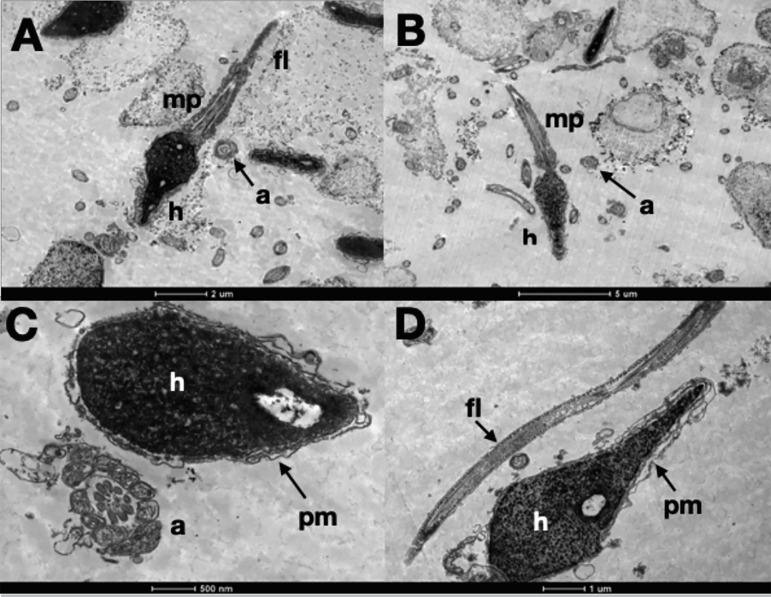
Transmission Electron Microscopy of Cryopreserved Sperm. A and C: Lyophilized in Sperm Freeze Medium: ruptured plasma membrane in head and flagellum, heterogeneous chromatin. Axoneme degenerated. B and D: Lyophilized in Freeze Medium: nuclei severed compromised, ruptured plasma membrane in head and flagellum, heterogeneous chromatin. a: axoneme, fl: flagellum, h: head, mp: midpiece, pm: plasma membrane.

After rehydration, lyophilized samples were initially analysed using optical microscopy and all spermatozoa were immotile. Analysis by Transmission Electron Microscopy showed that spermatozoa heads had ruptured plasma membranes, absence of acrosomes, nuclei with heterogeneous and decompressed chromatin. Mitochondria were deteriorated in the midpiece. Longitudinal columns of dense fibers were absent in the flagellum. Axonemes, in cross-sections, were disrupted with disorganized structures (Figure 3).

## DISCUSSION

This study observed the ultrastructure of the lyophilized human spermatozoa using Transmission Electron Microscopy, with emphasis on the analysis of structural integrity of the plasma membrane, mitochondria, acrosome, mid piece, axoneme, nucleus and its chromatin, and flagellum, after lyophilization using a fixed protocol.

When compared to the frozen/thawed samples (control group), the fresh samples showed higher concentration (75.74×10^6^ X 44.75x10^6^), and higher sperm progressive and non-progressive motility, 59% X 23.6% and 26.05% X 10.65%, respectively. All these occurred within the expected range, and can be explained by the fact that cryopreservation leads to a decrease in oxidative phosphorylation (Hallak *et al*., 2000; Wang *et al*., 2003). The morphological analysis using Transmission Electron Microscopy demonstrated that frozen spermatozoa maintained intact plasma, nucleus, mid piece and flagellum structures.

The observed effect of lyophilization on human sperm was the total loss of motility and severe injury to the cells with decreased viability after rehydration. These results are similar to those described by Kusakabe *et al.* (2008) and Gianaroli *et al*. (2012) who observed that although spermatozoa were physiologically dead, they still had intact DNA. Zhu *et al*. (2016) also observed head and tail membrane damage, however the authors used only SEM. These findings could be explained by the fact that glycerol-containing media cannot be lyophilized successfully, which probably led to the vast organelle damage (Alcay *et al*., 2015). *Freeze Medium* (Irvine Scientific) used in our study is rich in low density lipoprotein, whose function is to protect plasma membrane’s phospholipids against freezing and thawing damage. Samples that were lyophilized using this medium did not form a good lyophile, probably due to the presence of glycerol, and consequently suffered membrane rupture, despite the presence of low-density lipoproteins. These observed lesions were probably caused during the dehydration process, since in control frozen samples the structures and organelles remained intact.

Several solutions for lyophilization have been used to try to maintain sperm integrity, such as albumin (Wakayama & Yanagimachi, 1998), EDTA (Kaneko & Nakagata, 2006) trehalose (McGinnis *et al*., 2005), combinations of disaccharides such as lactose, trehalose and sucrose (Garcia *et al.,* 2014). Kaneko *et al*. (2003) observed that when lyophilization was performed in a slightly alkaline solution (pH 8.0) the integrity of the chromosomes was maintained, and embryo development was better. Although we used freeze mediums with a lower pH (7.4) we cannot state that this particularly caused to the high structural damage observed (Kaneko *et al.,* 2003).

As we used a constant pressure of 0.05 mbar throughout the lyophilization process, we consider this might have contributed to the sperm structural damage observed in our study. Kawase *et al*. (2007) recommend a primary pressure of 0.37 mbar for lyophilization of mouse spermatozoa. They suggested this value based on the observation of sperm DNA integrity and embryo development in vivo and in vitro. Kusakabe *et al*. (2008) used the pressure at 22 to 42x10^-3^ mbar for 4 hours to lyophilize samples of human semen. Gianaroli *et al*. (2012) used a similar pressure (22x10^-3^ mbar) overnight to lyophilize human semen.

The lyophilization process in our study lasted 24 hours. This long time interval may have led to excessive dehydration of the sample, and consequent damage to the sperm. Kwon *et al*. (2004) observed that increasing the time interval from 4 to 24 hours of lyophilization of pig spermatozoa gradually reduced embryonic development after fertilization. However, Gianaroli *et al*. (2012) lyophilized human semen overnight and obtained satisfactory results.

The storage temperature of the freeze-dried samples in our study ranged from 4º to 8ºC and was similar to what others had described. Wakayama & Yanagimachi (1998) demonstrated that refrigerated lyophilized mice sperm could generate healthy offspring after ICSI). Kaneko & Nakagata (2005) kept lyophilized murine sperm at 4ºC and observed normal DNA structure. Gianaroli *et al*. (2012) stored human sperm samples after lyophilization at 4ºC and also observed no damage to sperm DNA.

Liu *et al*. (2004) studied the behaviour of lyophilized rabbit sperm in EGTA, NaCl and Tris-HCl buffered medium using Scanning Electron Microscopy. They observed that many spermatozoa had broken or absent flagellum. Plasma membrane was ruptured in flagellum region and midpiece, alongside damaged axoneme structure, in accordance with our findings using Transmission Electron Microscopy. Wakayama & Yanagimachi (1998) freeze-dried mice sperm using culture media CZB and DMEM, and observed through Scanning Electron Microscopy, the following alterations in the sperm head: absence of the main segment of the acrosome, internal and external membranes of the acrosome ruptured or even absent. The nucleus remained intact and live offspring were obtained by performing ICSI with rehydrated spermatozoa. In our study we observed a decondensed chromatin, plasma membrane rupture and absent acrosome.

Martins *et al*. (2007) used three different solutions to lyophilize bovine sperm. The plasma membrane of spermatozoa was damaged in the lyophilization process independent of the medium used however the acrosome and mitochondria structures remained intact in all three media used. Differently from the study by Martins *et al*. (2007), the present study showed extensive damage to these structures.

Lyophilization might be an alternative to the use of liquid nitrogen for sperm cryopreservation since storage and transportation are a lot simpler, and the costs involved will be reduced. Also, as lyophilization is associated to virus inactivation, the risk of viral contamination will be virtually eliminated (Unger *et al*., 2009). More studies are needed to implement lyophilization as a routine technique in assisted reproduction clinics. Parameters such as temperature, pressure, time and lyophilization medium should be optimized to obtain lyophilized samples with intact DNA. After defining the best parameters, chromosomal viability tests should be performed.

To our knowledge, our study demonstrated, for the first time, the structure of the human spermatozoa after lyophilization using Transmission Electron Microscopy. The use of a fixed lyophilization protocol with media containing cryoprotectants might explain the damage to the structures. More studies are necessary to improve the results of sperm lyophilization. In the future, the use of lyophilization of spermatozoa might reduce the costs of fertility preservation, since there will be no need for storage space and transportation is simpler.
